# Fungi Unearthed: Transcripts Encoding Lignocellulolytic and Chitinolytic Enzymes in Forest Soil

**DOI:** 10.1371/journal.pone.0010971

**Published:** 2010-06-04

**Authors:** Harald Kellner, Micheline Vandenbol

**Affiliations:** Unité de Biologie Animale et Microbienne, Gembloux Agro-Bio Tech, Université de Liège, Gembloux, Belgium; Research Institute for Children and the Louisiana State University Health Sciences Center, United States of America

## Abstract

**Background:**

Fungi are the main organisms responsible for the degradation of biopolymers such as lignin, cellulose, hemicellulose, and chitin in forest ecosystems. Soil surveys largely target fungal diversity, paying less attention to fungal activity.

**Methodology/Principal Findings:**

Here we have focused on the organic horizon of a hardwood forest dominated by sugar maple that spreads widely across Eastern North America. The sampling site included three plots receiving normal atmospheric nitrogen deposition and three that received an extra 3 g nitrogen m^−2^ y^−1^ in form of sodium nitrate pellets since 1994, which led to increased accumulation of organic matter in the soil. Our aim was to assess, in samples taken from all six plots, transcript-level expression of fungal genes encoding lignocellulolytic and chitinolytic enzymes. For this we collected RNA from the forest soil, reverse-transcribed it, and amplified cDNAs of interest, using both published primer pairs as well as 23 newly developed ones. We thus detected transcript-level expression of 234 genes putatively encoding 26 different groups of fungal enzymes, notably major ligninolytic and diverse aromatic-oxidizing enzymes, various cellulose- and hemicellulose-degrading glycoside hydrolases and carbohydrate esterases, enzymes involved in chitin breakdown, *N*-acetylglucosamine metabolism, and cell wall degradation. Among the genes identified, 125 are homologous to known ascomycete genes and 105 to basidiomycete genes. Transcripts corresponding to all 26 enzyme groups were detected in both control and nitrogen-supplemented plots.

**Conclusions/Significance:**

Many of these enzyme groups are known to be important in soil turnover processes, but the contribution of some is probably underestimated. Our data highlight the importance of ascomycetes, as well as basidiomycetes, in important biogeochemical cycles. In the nitrogen-supplemented plots, we have detected no transcript-level gap likely to explain the observed increased carbon storage, which is more likely due to community changes and perhaps transcriptional and/or post-transcriptional down-regulation of relevant genes.

## Introduction

Fungi are an important and diverse component of soil microbial communities. They provide essential ecosystem services, such as decomposing organic matter, nutrient cycling, and in the case of mycorrhizal species, also nutrient transfer to plants [1]. In forest ecosystems they are largely responsible for breakdown of the abundant large biopolymers cellulose, hemicellulose, lignin, and chitin [2]. Activities of single fungal species or groups are difficult to access in soils. Methods commonly applied in soil surveys, such as determination of enzyme activities or soil respiration rates, phospholipid fatty acid analysis, or isolation of fungi, do not reveal which fungi in particular are responsible for diverse soil or ecosystem processes. In fact, they often only distinguish between prokaryotes and eukaryotes. A molecular approach is to use DNA extracted from soil to investigate fungal diversity, mainly *via* either the amplification of partial ribosomal genes [3]–[5], or the deduction of putative soil functions from protein-encoding fungal genes of single species or communities [6]–[9]. Only recently has it become possible to detect fungal activity on the basis of transcript-level gene expression. Used in studies focusing on single genes such as laccase, polyketide synthase, etc. [10]–[13], or on multiple genes expressed in parallel within communities [10], [14], this approach has revealed in forest environments the presence of ascomycetes and basidiomycetes with diverse ecological behaviors.

To elucidate the roles that fungi play in carbon sequestration and ecosystem functioning, there remains much to be learned about their contribution to biopolymer degradation and biogeochemical cycling. A problem that received considerable attention is the link between carbon cycling and nitrogen availability. Additional nitrogen can stimulate early-stage decomposition of plant litter and soil organic matter, but it suppresses this activity at later stages, when humus and lignin are abundant [15]. Northern-hemisphere temperate and boreal forest ecosystems cover large areas, representing huge terrestrial carbon stocks and acting as a substantial carbon sink (0.6–0.7 pg carbon yr^−1^) [16]. In a previous ecosystem study it was demonstrated that a decade of simulated additional atmospheric nitrogen deposition, at a rate expected by 2050 (additional 3 g nitrogen m^−2^ y^−1^ to ambient deposition), slowed the decay of plant detritus, thereby significantly increasing soil carbon storage in a temperate forest dominated by sugar maple (*Acer saccharum* Marsh.), that spreads widely across Eastern North America [17], [18]. Over the same period a decline in lignocellulolytic enzyme activity was observed in the forest floor [19].

One cause of this nitrogen-supplementation-induced slowing of plant detritus decomposition might be that single biodegradation steps involving important lignocellulolytic enzymes are “switched off”, leaving gaps in the degradative carbon cycle. Accumulating intermediates might then participate in negative feedback loops affecting other genes. It is known, for instance, that the expression of fungal genes required for cellulose biodegradation is subject to regulations such as catabolic repression [20]. Alternatively, increased nitrogen deposition might gradually down-regulate the expression of fungal genes encoding biopolymer-degrading enzymes, or the fungal community might change in response to additional nitrogen.

Here we have focused on this same sugar-maple-dominated forest site, with the intention of identifying transcriptionally expressed fungal genes encoding key lignocellulolytic, chitinolytic, and related enzymes. For this, we have isolated total RNA from the forest soil, reverse-transcribed it, and synthesized cDNAs using long-distance PCR (LD PCR), thus providing templates for subsequent detection of relevant transcripts by PCR. As few primers are available for accessing such genes, our first goal was to develop molecular tools for detecting transcripts encoding a wide range of fungal enzymes (phenol oxidases, peroxidases, cellulases, hemicellulases, esterases and chitinases…) that are both ecologically interesting and potentially useful in biotechnology [20]. Having developed these tools, we then addressed the following questions: i) Are transcripts of the targeted genes detectable in these soils? ii) Which fungi or fungal groups, i.e. ascomycetes or basidiomycetes, deploy them? iii) How does nitrogen supplementation affect the presence of transcript-level expression of the targeted genes and thereby the carbon balance of this ecosystem?

## Results and Discussion

### Transcript-level expression of ligninolytic, cellulolytic, chitinolytic, and related fungal enzymes

Twenty-three degenerate primer pairs were developed for PCR-based detection of transcripts related to biopolymer degradation in the organic horizon of the above-mentioned forest site (Supplementary Table S1). Published primers were also used: primers for fungal laccase and cellobiohydrolase genes, which were used in several soil surveys to gain first insights into molecular fungal diversity and putative activity in soils [6]–[8], [11], and recently published primer pairs for Class II fungal secretory heme peroxidase genes [21]. Some of the newly developed primer pairs appeared quite specific, others less so. Future improvements might include decreased primer degeneracy, a search for other conserved protein stretches useful for primer design, and adapting the PCR conditions. All primer pairs, however, proved useful in achieving our research goals.

A total of 234 partial genes were amplified from six forest soil cDNAs, corresponding to 26 different fungal enzyme groups involved in biopolymer degradation (Table 1, Supplementary Table S2). Twenty-three of these enzyme groups were accessed thanks to our newly developed primer pairs for fungal phyla or groups (Table S1).

Among the enzyme groups highlighted, 7 are involved directly or indirectly in the breakdown or conversion of lignin or in the oxidation of aromatic derivatives. Lignin has a complex three-dimensional structure based on phenyl propane units, and provides structural rigidity in woody plants [22] and protects energy-rich cellulose from degradation. Fungi are the main agents responsible for the decomposition of lignin, and transcripts were detected for manganese peroxidases [23], laccases [24], and cellobiose dehydrogenases (important in both lignin and cellulose degradation - see Table 1 and [25]). These enzyme groups are viewed as the three major ones acting on lignin [26] (FOLy database: LO1 – LO3, http://foly.esil.univ-mrs.fr/). We further detected transcripts of aromatic-oxidizing enzymes, including aromatic peroxygenases [27] and chloroperoxidases [28], which are members of a newly discovered group of heme-thiolate haloperoxidases displaying a broad range of extracellular enzymatic activities (see Table 1 and [29]). The involvement of this enzyme group in soil turnover processes has received relatively little attention, but their versatility is likely to make them an important focus of future studies. Another underestimated but relevant enzyme for which transcripts were found in our soil samples is fungal tyrosinase, which oxidizes monophenolic compounds [30]. Although this type of enzyme may contribute to the soil phenoloxidase activity measured with substrates such as L-3,4-dihydroxyphenylalanine [31], [32], there is a tendency to underestimate its contribution.

Fungal tyrosinases probably act mostly intracellularly, e.g. in pigmentation or detoxification processes, however there remains a need to investigate their contribution to extracellular phenol or lignin conversions in soils. Interestingly, the expression of a specific fungal tyrosinase was recently monitored in a forest soil, suggesting its potential importance [10]. Fungi need detoxifying enzymes to destroy potentially cytotoxic intermediates of, or participants in, lignin degradation. Laccases, tyrosinases, and also various peroxidases are thus suggested to have detoxifying effects [24], [32]. Another potentially detoxifying mechanism is ring cleavage of catecholate derivatives into citric acid intermediates by intradiol ring cleavage dioxygenases (IRDC) such as catechol 1,2-dioxygenase, also highlighted in this soil RNA study. Few fungal IRDCs have been characterized, but a 1,2,4-trihydroxybenzene 1,2-dioxygenase of *Phanerochaete chrysosporium*, that catalyzes key steps in the degradation pathway of vanillate, an intermediate in general lignin breakdown, suggests a contribution to soil turnover processes involving fungi [33]. The seventh type of lignolysis-related enzyme highlighted in this study is oxalate decarboxylase, an enzyme required by fungi to degrade oxalic acid, which is useful in lignin degradation as a chelator of Mn^3+^ ions [34] but cytotoxic when present in excess.

Enzymes related to the degradation of cellulose and hemicellulose, the most abundant biopolymers in terrestrial ecosystems [22], were also identified on the basis of the cDNAs amplified.

Cellulose, a linear polymer consisting of D-glucose monomers linked by β-1,4-glycosidic bonds, is the major structural component of cell walls in woody plants. To degrade cellulose and hemicellulose, fungi use a panoply of glycoside hydrolases (GH) and carbohydrate esterases (CE) [25], [35], [36]. Our work brought to light no less than twelve different glycoside hydrolase families and one type of carbohydrate esterase (Table 1, http://www.cazy.org [37]). Among these families, the enzymes endoglucanase, cellobiohydrolase, and β-glucosidase cover all essential steps of cellulose degradation down to monomeric glucose units [25]. Previous studies have revealed sequences corresponding to fungal endoglucanase and cellobiohydrolase in DNA extracted from decaying plant material and soil samples, thus highlighting their potential ecosystem importance [6], [38]. As for cellobiose dehydrogenase, mentioned above in relation to lignin degradation, its role in cellulose breakdown may be to control the quantity of the breakdown intermediate cellobiose, which in excess can repress cellobiohydrolase expression [25]. Furthermore, the generated hydroxyl radicals may act directly on cellulose polymers [25].

Hemicellulose, another major component of forest soil inputs from wood and leaves, is a frequently branching polymer with a heterogeneous composition. Its building blocks are mainly pentoses, hexoses, hexuronic acids, and deoxyhexoses [22]. Decaying leaf litter, for example, consists of 6.5–6.6% arabinose, 3.3–3.9% galactose, 1.2–4.9% mannose, 0.4–1.7% rhamnose, and 3.6–6.7% xylose [39]. Given this complexity, fungi deploy a distinct set of hydrolytic enzymes for hemicellulose decomposition [40]. In our study (Table 1) we detected putative transcripts of enzymes decomposing the xylan backbone and enzymes preferentially hydrolyzing xylan sidechains consisting of arabinan or glucuronic acid. Also identified was acetylxylan esterase involved in hydrolysis of acetyl groups of polymeric xylan and acetylated xylose. Putative transcripts for enzymes involved in the decomposition of mannan were also identified, as were transcripts for α-glucosidase, involved in starch hydrolysis.

Lastly, our study highlighted six groups of chitinolytic or related enzymes (Table 1). Chitin, the most abundant aminopolysaccharide in nature, is composed of *N*-acetyl-D-glucosamine monomers linked *via* β-1,4-glycosidic bonds [22]. In soils, chitin polymers are derived mainly from fungal sources but also from arthropods. They provide a considerable pool of nitrogen for other organisms. Several soil microorganisms, especially fungi use mainly hydrolytic enzymes to degrade chitin. It is unknown whether fungi preferentially use chitinase and related enzymes to attack other chitin-containing organisms, to recycle their own chitin structures, or both. The transcripts we registered (Table 1) concerned the chitinolytic enzymes chitinase and β-*N*-acetylhexosaminidase (expression of a specific fungal β-*N*-acetylhexosaminidase was recently monitored in a forest soil, indicating its relevance to soil processes and fungal nutrient acquisition [10]), fungal enzymes involved in other cell wall degradation or attack mechanisms (putative endo-α-1,4-polygalactosaminidase, degrading polygalactosamine polymers, and putative glucan endo-1,6-β-glucosidase, probably involved in microbial degradation of glucans that do not necessarily contain nitrogen), and also enzymes likely to be involved in *N*-acetyl-D-glucosamine metabolism in fungi (amidohydrolase or putative *N*-acetylglucosamine-6-phosphate deacetylase). Interestingly, we also amplified cDNA corresponding to the hydrolysis of nitrogen-rich *S*-formylglutathione.

In this study we have looked only at transcript-level gene expression in the soil horizon, so our data can tell us nothing about post-transcriptional regulation. It should be stressed, however, that many of the enzyme activities highlighted here have been detected with various classical enzymatic substrates in soil extracts from the organic horizon of this research site [19]. We thus feel confident that our transcript-level data provide a good picture of corresponding soil enzymatic activities. Final evidence might be provided by metaproteomic studies accessing all the enzymes present in soils and matching them with species or groups in databases [41]. To link community structure with transcript-level expression, it might be interesting to use high-throughput approaches which access the total soil metatranscriptome [42].

### Ascomycetes *vs.* basidiomycetes

How different kinds of fungi contribute to soil turnover processes is a major question in forest soil ecology. Basidiomycetes are regarded as major degraders of wood resources and are characterized as white-rot fungi decaying preferentially lignin components or brown-rot fungi decaying primarily cellulose [43]. Forest floor and soil horizons are much more heterogeneous than solid wood. They harbor different and ecologically more diverse fungal populations, notably comprising mycorrhizal, saprotrophic, and pathogenic/parasitic fungi. A current debate centers on the respective contributions of saprotrophic fungi *vs*. ectomycorrhizal basidiomycetes to decomposition and soil cycling in forest soils [44], [45]. Recent molecular biological data highlight the presence of varying proportions of ascomycetes in soils [3]–[5], but their actual activities remain largely unknown. In our survey, 125 of the highlighted genes are homologous to known ascomycete genes, 105 to putative basidiomycete genes, and a few to zygomycete or animal genes (Table S2). As this identification is based on a blastp search with sometimes low identity rates (Table S2), our results might change slightly with increasing support from database references and potentially higher identities. Furthermore, our primers do not necessarily target all fungal phyla (e.g. our cellobiose dehydrogenase primers are basidiomycete-specific, the β-glucosidase primers are ascomycete-specific, etc., see Table S1). Nevertheless, the high number of biogeochemical relevant ascomycete transcripts is new and noteworthy, as ligninolytic enzymes, such as laccase, tyrosinase, intradiol-ring cleavage dioxygenase, and potentially heme-thiolate haloperoxidase (Table 1, S2), are traditionally viewed as characteristic of basidiomycetes. The expression of ascomycete laccase or laccase-like multicopper oxidase has been reported for a beech forest organic horizon [11], and the presence of diverse ascomycete laccase genes has been found in decaying leaf samples from a salt marsh [9]. However, the full extent of the contribution of ascomycete laccases to lignin decomposition remains unclear, notably because these enzymes exert both polymerizing and depolymerizing activities. On the other hand, the involvement of ascomycetes in the decomposition of cellulose, hemicellulose, and chitin is well documented [46], and corresponds with our present findings (Table S2). Taken together, our data thus provide fundamental information on the expression of both ascomycete and basidiomycete genes encoding enzymes involved in the biogeochemical important processes of lignin, cellulose, hemicellulose, and chitin decomposition. As the number of annotated fungal genes in databases like GenBank increases, it should become possible to determine which fungi or ecological groups (e.g. ectomycorrhizal *vs*. saprotrophic basidiomycetes *vs*. saprotrophic ascomycetes) are most important in soil cycling processes.

### Fungal biogeochemical cycling under increased nitrogen deposition

Much debate centers on the mechanisms governing carbon sequestration in the globally important carbon sink constituted by the forests of the northern hemisphere [47]–[49]. The effect of anthropogenic nitrogen deposition from the atmosphere is variably believed to be strong or negligible [17], [50], [51], as depositions increase continuously over these forests [52]. The present research is part of a large-scale field experiment that has demonstrated a significant slowing of plant detritus decay in response to simulated atmospheric nitrogen deposition at a rate expected by 2050 in this region (additional 3 g nitrogen m^−2^ y^−1^ to ambient deposition). This means increased carbon storage in the soil of a temperate forest spreading widely across Eastern North America [17], [18]. In parallel, a decline in ligninolytic and cellulolytic enzyme activities has been observed in the forest floor [19]. Under laboratory conditions, high inorganic nitrogen concentrations can repress transcription of lignocellulolytic genes [53], [54]. This raises the question: might anthropogenic nitrogen deposition elicit a similar effect?

In the above-mentioned field-based experiment, replicate plots continuously received either ambient or simulated increased atmospheric nitrogen deposition over a 14-yr period beginning in 1994. The simulated deposition treatment consisted of 3 g sodium nitrate m^−2^ y^−1^ applied to the forest floor in equal increments over the growing season [18]. PCR amplification was performed on cDNA from each plot, but cloning and sequencing of PCR products was performed only on pooled samples (Table S1).

As transcripts corresponding to all 26 enzyme groups highlighted here were found in both nitrogen-supplemented and control soils (Table 1, Table S2), our data provide no evidence of a total transcriptional “switch-off” in response to increased nitrogen deposition. Transcriptional down-regulation of relevant fungal genes cannot be excluded, but to assess its contribution would require large-scale application of real-time PCR as used for single specific genes from a forest soil [10]. We do provide evidence suggesting that increased nitrogen deposition induced changes in the composition of the fungal community: only 22 of the 234 detected transcripts were found in both nitrogen-supplemented and control plots, the remainder being potentially unique to one or the other. This finding is interesting, but it must be stressed that our sampling setup did not allow an in-depth community analysis. Our data may provide a starting point for an ulterior exhaustive analysis of the biopolymer-degrading fungal community. It should also be mentioned that our data represent only one sampling site of the large-scale field experiment. To draw reliable conclusions, one should use multiple data from all sites. Nevertheless, the results presented here provide the first molecular evidence of major fungal involvement in biogeochemical cycling, i.e. lignocellulose and chitin decomposition, even in a manipulated ecosystem.

### Conclusion

Fungi provide essential ecosystem services, even in a changing environment. Future research should aim to understand molecular regulatory mechanisms in soils in order to draw conclusions about effects on ecosystems. Some of the tools provided here may help to establish links between fungal communities, their enzymatic activities in soils, and the consequences for ecosystems. Many of the fungal enzymes highlighted in this work receive much attention in applied biotechnological research, and the molecular tools developed here may find further use in both basic and applied research.

## Materials and Methods

### Study site and soil sampling

Soil samples were taken from a long-term study site in Oceana County, Michigan, USA (43° 40′ N, 86° 09′ W), a northern hardwood forest dominated by *Acer saccharum* Marsh. This is the southernmost site of a zone involved in a long-term, 500-km climatic and nitrogen-deposition gradient study begun in 1994 [17], including four sites with varying amounts of ambient atmospheric nitrogen (N) deposition (0.68–1.17 g N m^−2^ yr^−1^, lowest amount in the northernmost site, highest in the southernmost site). At the Oceana site, three 30-m×30-m plots receive ambient atmospheric nitrogen deposition and three 30-m×30-m plots receive simulated increased atmospheric nitrogen deposition. The simulated atmospheric nitrogen deposition treatment (an additional 3 g N m^−2^ y^−1^) was initiated in 1994 and consists of six equal applications of sodium nitrate (NaNO_3_) delivered as dry pellets to the forest floor over the growing season. The soil is sandy, mixed, a mesic Entic Haplorthod. Soil sampling was done in November 2007 after leaf senescence. In each of the six plots, 10 random 0.1 m×0.1 m litter samples (intermediately decomposed organic horizon O_e_ and highly decomposed O_a_ horizon; together up to 2 cm thick) were collected, composited, and homogenized (by cutting the litter in 1–2 cm^2^ pieces and mixing them in a plastic container), in order to ensure plot coverage and representation of all overstory tree species. The homogenized samples were immediately transferred to liquid nitrogen for RNA extraction.

### RNA extraction and cDNA construction

A previously published protocol [13] was used to extract total RNA from the six composite forest floor samples obtained from the Oceana research site. Briefly, the RNA of one gram of forest floor was extracted with glass beads and a phenol-based solution. The sample was disrupted with the FastPrep FP120A instrument (MP Biomedicals, Solon, USA) for 30 s at a speed of 6.5. The RNA of this crude mix was then centrifuged, precipitated with ethanol, and separated with the RNA/DNA Midi kit (10) (Qiagen, Hilden, Germany) as recommended by the manufacturer. Before further purification of the RNA with the RNeasy Plant Mini kit (Qiagen), an extra DNAse step (Qiagen) was carried out as recommended by the manufacturer. Three microliters of purified DNA-free RNA was used as template for reverse transcription, after which the six cDNAs were amplified *via* 17 cycles of a long-distance PCR (LD-PCR) using the SMART™ PCR cDNA Synthesis Kit (Clontech, Mountain View, USA).

### Primers, PCR conditions, cloning, and sequencing

Degenerate primer pairs for amplifying coding sequences corresponding to fungal ligninolytic, cellulolytic, hemicellulolytic, and chitinolytic enzymes were developed on the basis of reference protein sequences from curated databases like CAZy (http://www.cazy.org [37], see also http://www.cazypedia.org) or FOLy (http://foly.esil.univ-mrs.fr/ [26]), or GenBank. The reference sequences were compared against the NCBI database standard protein-protein BLAST (blastp) (Table S1), and the distance tree option implemented in the NCBI result page was used to display the phylogenetic relationship of each protein of interest among different fungal groups. Then the implemented multiple alignment function for distinct clades was used to find conserved protein sequences of the selected candidates (Table S1). Using this procedure, degenerate primer pairs were developed for conserved protein regions of each enzyme group, and are able to amplify either from broad fungal groups in general, or fungal subsets for example ascomycetes or basidiomycetes, or members of fungal family level (Table S1). For PCR amplification, in a 25 µl PCR reaction using DreamTaq Green PCR Master Mix (Fermentas, Burlington, Canada), 0.25 µl of forward and reverse primer (100 mM, Eurogentec, Liege, Belgium) and 0.5 µl cDNA template were added. The following program on a PT-200 thermocycler (MJ Research, Watertown, USA) was used for amplification: initial denaturation for 5 min at 94°C, 35 cycles of denaturation (45 s at 94°C), annealing (45 s at 50°C), and elongation (1 min 40 s at 72°C), followed by a final elongation step for 10 min at 72°C. PCR products of expected sizes (Table S1) were gel purified, composited among three ambient and simulated increased nitrogen receiving plots, and cloned into the pCR4-TOPO vector using the TOPO TA Cloning kit for sequencing (Invitrogen Life Technology, Karlsruhe, Germany). About 10 positive clones for each expressed enzyme group and per treatment were sequenced at GATC Biotech AG (Konstanz, Germany). Obtained nucleotide sequences were edited with BioEdit 7 [55], translated to protein sequences, and identified with blastp (Table S1). Protein sequences were aligned and phylogenetically compared with references obtained from NCBI. They are accessible on request (http://www.haraldkellner.com/oceanastudy/welcome.html). All sequences were submitted to GenBank and are available under accession numbers FJ040216-FJ040219, FJ040222-FJ040225, GU734340-GU734565.

**Table 1:** Expressed genes encoding putative lignocellulolytic and chitinolytic fungal enzymes found in ambient and simulated increased nitrogen litter samples.

Enzyme (EC number)	Putative enzyme function	Total number of transcribed gene types (ambient, nitrogen plot)	Detected fungal phyla#
***Ligninolytic enzymes and related***			
Manganese peroxidase (EC 1.11.1.13), Class II of the non-animal heme peroxidase superfamily	Extracellular lignin oxidation and breakdown *via* Mn^3+^	4 (1, 3)	B
Laccase (EC 1.10.3.2)	Extracelluar oxidation of phenolics and lignin	9 (8, 6)	A, B
Cellobiose dehydrogenase (EC 1.1.99.18)	Extracellular; putative lignin oxidation *via* Fenton reaction (generation of hydroxyl-radicals)	4 (2, 3)	B
Oxalate decarboxylase (EC 4.1.1.2)	Oxalate breakdown	17 (6, 11)	A, B
***Enzymes oxidizing aromatics***			
Heme-thiolate peroxidases; i.e. aromatic peroxygenase (EC 1.11.2.-), chloroperoxidase (EC 1.11.1.10)	Extracellular oxygenations (*O*-dealkylation, hydroxylation, epoxidation, sulfoxidation, *N*-oxidation, etc.); unspecific halogenation	11 (9, 3)	A, B
Tyrosinase (EC 1.14.18.1)	Intracellular and cell-wall-associated oxidation of phenols, pigmentation	5 (3, 2)	A, B
Intradiol ring cleavage dioxygenase, putative catechol 1,2-dioxygenase (EC 1.13.11.1) or hydroxyquinol 1,2-dioxygenase (EC 1.13.11.37)	Intracellular cleavage of aromatic rings	7 (5, 3)	A
***Cellulolytic, hemicellulolytic and related enzymes (all extracellular)*** §			
GH3: putative β-glucosidase (EC 3.2.1.21) or xylan 1,4-β-xylosidase (EC 3.2.1.37)	Cellulose and xylan backbone degradation	25 (22, 4)	A
GH5: putative mannan endo-1,4-β-mannosidase (EC 3.2.1.78) or endoglucanase (EC 3.2.1.4)	Mannan & cellulose degradation	9 (4, 5)	A, B
GH6: cellulose 1,4-β-cellobiosidase, i.e. cellobiohydrolase II (EC 3.2.1.91)	Cellulose degradation	3 (2, 1)	A, B
GH7: cellobiohydrolase I (EC 3.2.1.-) or endoglucanase (EC 3.2.1.4)	Cellulose degradation	11 (5, 6)	A, B
GH10: endo-1,4-β-xylanase (EC 3.2.1.8)	Xylan backbone degradation	3 (2, 1)	A, B
GH11: endo-1,4-β-xylanase (EC 3.2.1.8)	Xylan backbone degradation	14 (10, 8)	A, B
GH31: α-glucosidase (EC 3.2.1.20)	Starch degradation	10 (4, 6)	A
GH45: endoglucanase (EC 3.2.1.4)	Cellulose degradation	11 (7, 7)	A, Z
GH51: α-L-arabinofuranosidase (EC 3.2.1.55)	Xylan sidechain (arabinan) degradation	9 (5, 4)	B
GH67: α-glucuronidase (EC 3.2.1.139) or xylan α-1,2-glucuronosidase (EC 3.2.1.131)	Xylan sidechain (glucuronic acid) degradation	8 (5, 3)	A
GH74: endoglucanase (EC 3.2.1.4) or putative xyloglucan-specific endo-β-1,4-glucanase (EC 3.2.1.151)	Cellulose degradation	4 (2, 2)	A, B
GH92: putative α-1,2-mannosidase (EC 3.2.1.-)	Mannan sidechain degradation	9 (2, 8)	A
CE1: acetylxylan esterase (EC 3.1.1.72)	Xylan sidechain degradation	5 (2, 3)	A
Cellobiose dehydrogenase (EC 1.1.99.18)	Cellobiose decomposition; generation of hydroxyl radicals putatively acting on cellulose	4 (2, 3)	B
***Enzymes related to chitinolysis and aminosugar metabolism***			
GH18: chitinase (EC 3.2.1.14)	Chitin degradation	12 (4, 9)	A, B
GH20: β-*N*-acetylhexosaminidase (EC 3.2.1.52)	Chitobiose hydrolysis	15 (9, 8)	A, B
GH30: putative glucan endo-1,6-β-glucosidase (EC 3.2.1.75)	Microbial glucan degradation (e.g. fungal cell walls)	2 (1, 1)	A
GH114: putative endo-α-1,4-polygalactosaminidase (EC 3.2.1.109)	Glucan degradation (e.g. fungal cell walls)	10 (6, 4)	A
CE1: *S*-formylglutathione hydrolase (EC 3.1.2.12)	Methane cycle (or nitrogen cycle)	8 (4, 5)	A
CE9: amidohydrolase (EC 3.5.-.-) or putative *N*-acetylglucosamine-6-phosphate deacetylase (EC 3.5.1.25)	Internal aminosugar metabolism, nitrogen cycle	9 (7, 3)	B

#Fungal phyla: A – ascomycetes, B – basidiomycetes, Z – zygomycetes.

§GH – glycoside hydrolase family and CE – carbohydrate esterase family according to www.cazy.org [37].

## Supporting Information

Table S1 Developed degenerate primer pairs for different fungal enzyme groups.
(0.12 MB RTF)

Table S2 Transcribed genes giving a blastp match, putative fungal phylum (A - ascomycetes, B - basidiomycetes, Z - zygomycetes), derived ambient (A) or nitrogen-amended (N) plots, and accession number.
(0.50 MB RTF)
