# Correction: Fungi Unearthed: Transcripts Encoding Lignocellulolytic and Chitinolytic Enzymes in Forest Soil

**DOI:** 10.1371/annotation/84b7b537-84f6-49e6-ac7c-9a2f0ad3f862

**Published:** 2010-09-29

**Authors:** Harald Kellner, Donald R. Zak, Micheline Vandenbol

Dr. Donald R. Zak should be included in the author byline instead of the Acknowledgments. He should be listed as the second author, and his affiliations are 2,3:

2 School of Natural Resources and Environment, University of Michigan, Ann Arbor, USA

3 Department of Ecology and Evolutionary Biology, University of Michigan, Ann Arbor, USA

The contributions of this author are as follows: conceived and designed the experiments, contributed reagents/materials/analysis tools, and wrote the paper.

The correct citation is: Kellner H, Zak DR, Vandenbol M (2010) Fungi Unearthed: Transcripts Encoding Lignocellulolytic and Chitinolytic Enzymes in Forest Soil. PLoS ONE 5(6): e10971. doi:10.1371/journal.pone.0010971

The funding section should include the following statement: This research was supported by grants from the U.S. National Science Foundation and the Office of Science (BER), U.S. Department of Energy. 

