# Supplementary material for: Fungi Unearthed: Transcripts Encoding Lignocellulolytic and Chitinolytic Enzymes in Forest Soil
Source: PLoS One. 2010 Jun 4;5(6):e10971. doi: 10.1371/journal.pone.0010971 (PMC2881045; doi:10.1371/journal.pone.0010971)
Supplement: Table S1 — Developed degenerate primer pairs for different fungal enzyme groups. (0.12 MB RTF) [file pone.0010971.s001.rtf]

Supplementary Table 1: Developed degenerate primer pairs for different fungal enzyme groups

Enzyme (EC number)	Primers 5'-3', forward / reverse	Conserved peptide, forward / reverse primer site	Reference used for blastp search	PCR product length (bp)	Potential primer specificity	Amplified PCR products; ambient / nitrate plots 	Clones yielding correct insert	
Ligninolytic enzymes, aromatics oxidizing enzymes and related	
Heme-thiolate peroxidase; i.e. aromatic peroxygenase (EC 1.11.2.-) or chloroperoxidase (EC 1.11.1.10); NCBI peroxidase subfamily 2	APO_65F: AAY GCI ATG GCN AAY CAY GG /
APO_130R: GC RTC RTG YTC IAT NCC	NAMANHG / GIEHDAS	Postia placenta XP_002474926	~210	basidiomycetes, few ascomycetes	3/3	>90%	
Tyrosinase (EC 1.14.18.1)	Tyr_137F: CCN TWY TGG GAY TGG GC /
Tyr_282R: TG RTG IAR RAA RAA DAT IGG	PFWDWA / PIFFLHH	Neurospora crassa OR74A, XP_964823	~470 & ~600	asco- & basidiomycetes	3/3	78%	
Intradiol ring cleavage dioxygenase superfamily, putative catechol 1,2-dioxygenase (EC 1.13.11.1)	IRDC_F: TAY CCI ATH CCI CAY GAY GG /
IRDC_R: TT DAC ICC RAA DAC IGC RTC	YPIPHDG / DAVFGVK	Botryotinia fuckeliana B05.10, XP_001551553	~175	ascomycete subgroups	3/3	91%	
Oxalate decarboxylase (EC 4.1.1.2), NCBI cupin 2 superfamily	OxDC_190F: GGI GAY YTI TGG TAY TTY CC /
OxDC_340R: GT IGG RTG CCA RTG IAR YTC	GDLWYFP / ELHWHPT	Flammulina velutipes, AAF13275	~450	mainly basidiomycetes	3/3	>90%	
								
Cellulolytic and hemicellulolytic enzymes, and related	
β-glucosidase (EC 3.2.1.21) or xylan 1,4-β-xylosidase (EC 3.2.1.37), GH3	Glc1_155F: GGI MGI AAY TGG GAR GGN TT /
Glc1_235R: AY IGC RTC IGC RAA NGG CCA	GRNWEGF / WPFADA(I/V)	Chaetomium thermophilum, ABR57325	~230	ascomycetes	3/3	>90%	
β-mannosidase (EC 3.2.1.25), GH5	GH5_130F: GI GTH TGG GGI TTY AAY GA /
GH5_245R: GG YTC RTT ISG IAR YTC CCA	RVWGFND / WEL(A/G)NEP	Penicillium marneffei ATCC 18224, XP_002153357	~350	asco- & basidiomycetes	3/3	100%	
Cellobiohydrolase II (EC 3.2.1.91), GH6	GH6_270F: GAY GCI GGI CAY GCN GGN TGG /
GH6_400R: TC NCC NCC IGG YTT DAC CCA	DAGHAGW / WVKPGGE	Acremonium cellulolyticus Y-94, BAA74458	~410	asco- & basidiomycetes	3/2	63%	
Endo-1,4-β-xylanase (EC 3.2.1.8), GH10	GH10_80F: CCI GAR AAY WSI ATG AAR TGG / 
GH10_190R: TA RTC RTT IAT RTA IAR YTT	PENSMKW / KLYINDY	Sclerotinia sclerotiorum 1980, XP_001598420	~390	asco- & basidiomycetes	2/3	100%	
Endo-1,4-β-xylanase (EC 3.2.1.8), GH11	GH11_80F: GGI AAR GGN TGG AAY CCN GG /
GH11_200R: TA ICC YTC IGT IGC DAC DAT	GKGWNPG / IVATEGY	Botryotinia fuckeliana B05.10, XP_001558008	~400	asco- & basidiomycetes	3/3	>90%	
α-glucosidase (EC 3.2.1.20), GH31	GH31_350F: CAY CAR TGY MGI TGG GGN TA /
GH31_660R: TT RTC ICC NCC CCA RTG NCC	HQCRWGY / GHWGGDN	Aspergillus terreus NIH2624, XP_001217611	~980	ascomycetes	3/3	81%	
Endoglucanase (EC 3.2.1.4), GH45	GH45_155F: GI TAY TGG GAY TGY TGY AA /
GH45_280R: CC IAC ICC NCC NCC NGG CAT	RYWDCCK / MPGGGVG	Pyrenophora tritici-repentis Pt-1C-BFP, XP_001935162	~370	asco- & zygomycetes	3/3	>90%	
α-L-arabinofuranosidase (EC 3.2.1.55), GH51	GH51_280F: AGN TGG CAR TGG AAY GCN AC /
GH51_350R: AT YTG RTC DAT IGC YTG YTG	RWQWNAT / QQAIDQI	Postia placenta Mad-698-R, EED80442	~225	basidiomycetes	3/2	94%	
α-glucuronidase (EC 3.2.1.139), GH67	GH67_390F: GGI CCI ATH GAY TTY CAR GT /
GH67_570R: GC ICK IGT CCA YTG NCC CCA	GPIDFQV / WGQWTRA	Penicillium chrysogenum Wisconsin 54-1255, CAP96716	~560	ascomycetes	3/3	90%	
Endoglucanase (EC 3.2.1.4), Xyloglucanase (EC 3.2.1.151), GH74	GH74_130F: TTY AAR GTI GGI GGN AAY ATG /
GH74_280R: CC RTC RTA IGG ICC NGC NCC	FKVGGNM / GAGPYDG	Coprinopsis cinerea okayama7#130, XP_001830215	~460	asco- & basidiomycetes	3/2	80%	
α-1,2-mannosidase (EC 3.2.1.-), GH92	GH92_350F: ACI GGI GAR AAY CCN YTN TGG /
GH92_420R: GC RTT ISW ICC ICC YTG NGT	TGENPLW / TQGGSNA	Neurospora crassa OR74A, XP_960424	~220	ascomycetes	3/3	93%	
Acetyl xylan esterase (EC 3.1.1.72), CE1; NCBI esterase-lipase superfamily	AXE_155F: GTH ATG GCI GCI ACN TAY CC /
AXE_250R: TA ICC RAA DAC ICC NGC CCA	VMAATYP / WAGVFGY	Neurospora crassa OR74A, XP_964544	~290	ascomycetes	3/3	90%	
Cellobiose dehydrogenase (EC 1.1.99.18)	CDH320F: GGI GCN YTI TAY TGG TAY CC / 
CDH500R: CAT RTC IGT IGG ICC DAT NCC	GALYWYP / GIGPTDM	Athelia rolfsii, AAO64483	~530	basidiomycetes	3/3	100%	
								
Chitinolytic enzymes, aminosugar metabolism and related	
Chitinase (EC 3.2.1.14), GH18	Chit150F: GAY TTY GAY TGG GAR TAY CC /
Chit250R: AR IGG IGC RTT IGG NCC DAC	DFDWEYP / VGPNAPL	Postia placenta Mad-698-R, XP_002469877	~280	basidiomycetes, few ascomycetes	3/3	>90%	
β-hexosaminidase (EC 3.2.1.52), GH20	GH20_340F: ACI GGI GGN GAY GAR ATH AA /
GH20_440R: CC RCA RTC IAR RTA RAA RTA	TGGDEIN / YFYLDCG	Coprinopsis cinerea okayama7#130, XP_001835638	~305	basidiomycetes	3/3	>90%	
β-1,6-glucanase (EC 3.2.1.75), GH30	GH30_180F: CCI GGN TGG ATG AAR YTN AA /
GH30_330R: CCA RCA YTC NGT CAT RTA YTG	PGWMKLN / QYMTECW	Talaromyces stipitatus ATCC 10500, XP_002486481	~460	ascomycetes	3/3	67%	
endo-α-1,4-polygalactosaminidase (EC 3.2.1.109), GH114; NCBI DUF297 superfamily	GH114_120F: ATI TGY TAY TTY WSI GCN GG /
GH114_270R: TC DAT RTG RAA DAC IGG YTT	ICYFSAG / KPVFHIE	Phaeosphaeria nodorum SN15, XP_001804422	~450	ascomycetes	3/3	79%	
S-formylglutathione hydrolase (EC 3.1.2.12), CE1; NCBI esterase-lipase superfamily	CE1_150F: ATG GGI GGI CAY GGN GCN YT /
CE1_220R: AA RTT RTC ICC IGT NCC DAC	MGGHGAL / VGTGDNF	Pyrenophora tritici-repentis Pt-1C-BFP, XP_001937330	~235, but multiple gel bands	ascomycetes	3/3	80%	
Putative N-acetylglucosamin 6-phospat deacetylase (EC 3.5.1.25), CE9; NCBI metallo dependent hydrolase superfamily	CE9_355F: CAY TGY TAY GAR GCI GTH GA /
CE9_430R: AC IGG RTG RTC ISW YTT CAT	HCYEAVD / MKSDHPV	Coprinopsis cinerea okayama7#130, XP_001830115	~260	basidiomycetes	3/3	>90%	
