# Supplementary material for: Fungi Unearthed: Transcripts Encoding Lignocellulolytic and Chitinolytic Enzymes in Forest Soil
Source: PLoS One. 2010 Jun 4;5(6):e10971. doi: 10.1371/journal.pone.0010971 (PMC2881045; doi:10.1371/journal.pone.0010971)
Supplement: Table S2 — Transcribed genes giving a blastp match, putative fungal phylum (A - ascomycetes, B - basidiomycetes, Z - zygomycetes), derived ambient (A) or nitrogen-amended (N) plots, and accession number. (0.50 MB RTF) [file pone.0010971.s002.rtf]

Supplementary Table 2: Transcribed genes giving a blastp match, putative fungal phylum (A – ascomycetes, B – basidiomycetes, Z – zygomycetes), derived ambient (A) or nitrogen-amended (N) plots, and accession number

Enzyme	Accession number	NCBI blastp results (top hit), % identity, E value	NCBI blastp results (closest annotated functional hit if best hit was hypothetical), % identity, E value	Putative fungal phylum	Ambient/ Nitrogen	
Laccase - type 1	FJ040222	EEH11598, laccase [Ajellomyces capsulatus G186AR], 73%, 2e-12		A	A,N	
Laccase - type 2	FJ040223	ABO38534, laccase-like multicopper oxidase [Piloderma croceum], 85%, 6e-08		B	A,N	
Laccase - type 3	FJ040224	CAD65792, laccase [Mycena zephirus], 78%, 6e-11		B	A,N	
Laccase - type 4	FJ040225	CAR48258, phenol oxidase [Pleurotus ostreatus], 89%, 1e-20		B	A,N	
Laccase - type 5.1	GU734541	ACL13716, laccase-like multicopper oxidase [Rhodocollybia maculata], 100%, 3e-24		B	A	
Laccase - type 5.2	GU734542	ACL13716, laccase-like multicopper oxidase [Rhodocollybia maculata], 97%, 6e-24		B	A,N	
Laccase - type 6	GU734543	CAD12464, laccase [Pleurotus cornucopiae], 87%, 6e-10		B	A	
Laccase - type 7	GU734544	CAD12464, laccase [Pleurotus cornucopiae], 84%, 3e-09		B	A	
Laccase - type 8	GU734545	CAE81289, laccase [Rigidoporus microporus], 76%, 3e-15		B	N	
Mn-Peroxidase - type 1	GU734546	AAO61784, peroxidase [Ceriporiopsis subvermispora], 67%, 1e-06		B	A	
Mn-Peroxidase - type 2	GU734547	ACM47220, manganese peroxidase 3 [Pleurotus ostreatus], 69%, 3e-33 		B	N	
Mn-Peroxidase - type 3	GU734548	AAY42945, manganese peroxidase 3 [Pleurotus pulmonarius], 72%, 8e-42
		B	N	
Mn-Peroxidase - type 4	GU734549	AAY42945, manganese peroxidase 3 [Pleurotus pulmonarius], 73%, 1e-42		B	N	
Aromatic peroxygenase - type 1	GU734340	EDR10604, predicted protein [Laccaria bicolor S238N-H82], 72%, 1e-23	CAC03461, putative chloroperoxidase [Agaricus bisporus], 60%, 6e-17	B	A,N	
Aromatic peroxygenase - type 2	GU734341	EAU83861, hypothetical protein CC1G_09530 [Coprinopsis cinerea okayama7#130], 71%, 9e-24	CAC03461, putative chloroperoxidase [Agaricus bisporus], 69%, 8e-21	B	A	
Aromatic peroxygenase - type 3	GU734342	EED79857, chloroperoxidase-like protein [Postia placenta], 44%, 1e-07	AAA33025, chloroperoxidase [Leptoxyphium fumago], 32%, 2.9 	B/(A)	A	
Aromatic peroxygenase - type 4	GU734343	EED79857, chloroperoxidase-like protein [Postia placenta], 67%, 1e-25		B	N	
Aromatic peroxygenase - type 5	GU734344	EDR10604, predicted protein [Laccaria bicolor S238N-H82], 78%, 9e-28	CAC03461, putative chloroperoxidase [Agaricus bisporus], 61%, 9e-19	B	N	
Aromatic peroxygenase - type 6.1	GU734345	EDR10604, predicted protein [Laccaria bicolor S238N-H82], 73%, 6e-23	CAC03461, putative chloroperoxidase [Agaricus bisporus], 63%, 1e-17	B	A	
Aromatic peroxygenase - type 6.2	GU734346	EDR10604, predicted protein [Laccaria bicolor S238N-H82], 73%, 6e-23	CAC03461, putative chloroperoxidase [Agaricus bisporus], 63%, 1e-17	B	A	
Aromatic peroxygenase - type 7	GU734347	EAU83861, hypothetical protein CC1G_09530 [Coprinopsis cinerea okayama7#130], 80%, 2e-28	CAC03461, putative chloroperoxidase [Agaricus bisporus], 70%, 7e-22	B	A	
Aromatic peroxygenase - type 8	GU734348	EAU83861, hypothetical protein CC1G_09530 [Coprinopsis cinerea okayama7#130], 60%, 3e-21	CAC03461, putative chloroperoxidase [Agaricus bisporus], 61%, 3e-19	B	A	
Aromatic peroxygenase - type 9	GU734349	EAU83861, hypothetical protein CC1G_09530 [Coprinopsis cinerea okayama7#130], 55%, 4e18	CAC03461, putative chloroperoxidase [Agaricus bisporus], 47%, 1e-12	B	A	
Aromatic peroxygenase - type 10	GU734350	EAU83861, hypothetical protein CC1G_09530 [Coprinopsis cinerea okayama7#130], 57%, 4e-19	CAC03461, putative chloroperoxidase [Agaricus bisporus], 60%, 1e-18	B	A	
Intradiol ring cleavage dioxygenase - type 1	GU734351	EDO02947, hypothetical protein SS1G_05424 [Sclerotinia sclerotiorum 1980], 93%, 8e-27	EAL90759, catechol dioxygenase, putative [Aspergillus fumigatus Af293], 84%, 4e-23	A	A,N	
Intradiol ring cleavage dioxygenase - type 2	GU734352	EDN30607, hypothetical protein BC1G_09927 [Botryotinia fuckeliana B05.10], 94%, 6e-27	EAL90759, catechol dioxygenase, putative [Aspergillus fumigatus Af293], 84%, 7e-23	A	A	
Intradiol ring cleavage dioxygenase - type 3	GU734353	EDN30607, hypothetical protein BC1G_09927 [Botryotinia fuckeliana B05.10], 93%, 2e-26	EEH34378, hydroxyquinol 1,2-dioxygenase [Paracoccidioides brasiliensis Pb01], 79%, 2e-22	A	A	
Intradiol ring cleavage dioxygenase - type 4	GU734354	EDN30607, hypothetical protein BC1G_09927 [Botryotinia fuckeliana B05.10], 93%, 1e-26	EAL90759, catechol dioxygenase, putative [Aspergillus fumigatus Af293], 87%, 5e-23	A	N	
Intradiol ring cleavage dioxygenase - type 5	GU734355	EDO02947, hypothetical protein SS1G_05424 [Sclerotinia sclerotiorum 1980], 84%, 7e-24	EAL90759, catechol dioxygenase, putative [Aspergillus fumigatus Af293], 87%, 5e-23	A	A	
Intradiol ring cleavage dioxygenase - type 6	GU734356	CAP93956, Pc16g12860 [Penicillium chrysogenum Wisconsin 54-1255], 91%, 1e-24	CAF32141, dioxygenase, putative [Aspergillus fumigatus], 91%, 1e-24	A	A	
Intradiol ring cleavage dioxygenase - type 7	GU734357	EDO02947, hypothetical protein SS1G_05424 [Sclerotinia sclerotiorum 1980], 91%, 5e-26	EAL90759, catechol dioxygenase, putative [Aspergillus fumigatus Af293], 84%, 3e-23	A	N	
Cellobiose dehydrogenase - type 1	FJ040216	BAC20641, cellobiose dehydrogenase [Grifola frondosa], 82%, 1e-80		B	A,N	
Cellobiose dehydrogenase - type 2	FJ040217	BAC20641, cellobiose dehydrogenase [Grifola frondosa], 70%, 6e-67		B	A	
Cellobiose dehydrogenase - type 3	FJ040218	BAD36748, cellobiose dehydrogenase [Irpex lacteus], 69%, 8e-66		B	N	
Cellobiose dehydrogenase - type 4	FJ040219	EEB90497, hypothetical protein MPER_11289 [Moniliophthora perniciosa FA553], 81%, 4e-80	BAD36748, cellobiose dehydrogenase [Irpex lacteus], 71%, 7e-70	B	N	
Oxalate decarboxylase type - 1	GU734358	EED81598, candidate oxalate decarboxylase [Postia placenta Mad-698-R], 61%, 4e-49	CAD91552, oxalate oxidase [Ceriporiopsis subvermispora], 52%, 2e-43	B	A	
Oxalate decarboxylase type - 2	GU734359	EEB89037, hypothetical protein MPER_12919 [Moniliophthora perniciosa FA553], 79%, 7e-63	AAO89278, oxalate decarboxylase [Flammulina sp. IJF 140502], 63%, 4e-49	B	N	
Oxalate decarboxylase type - 3	GU734360	EDQ99885, predicted protein [Laccaria bicolor S238N-H82], 60%, 1e-48	CAD91553, oxalate oxidase [Ceriporiopsis subvermispora], 61%, 1e-46	B	N	
Oxalate decarboxylase type - 4.1	GU734361	EAU85209, hypothetical protein CC1G_06225 [Coprinopsis cinerea okayama7#130], 74%, 1e-59	AAO89278, oxalate decarboxylase [Flammulina sp. IJF 140502], 63%, 5e-55	B	N	
Oxalate decarboxylase type - 4.2	GU734362	EAU85209, hypothetical protein CC1G_06225 [Coprinopsis cinerea okayama7#130], 75%, 2e-60	AAO89278, oxalate decarboxylase [Flammulina sp. IJF 140502], 69%, 2e-55	B	A	
Oxalate decarboxylase type - 5.1	GU734363	EAU85209, hypothetical protein CC1G_06225 [Coprinopsis cinerea okayama7#130], 76%, 4e-63	AAO89278, oxalate decarboxylase [Flammulina sp. IJF 140502], 69%, 7e-56 	B	N	
Oxalate decarboxylase type - 5.2	GU734364	EAU85209, hypothetical protein CC1G_06225 [Coprinopsis cinerea okayama7#130], 76%, 4e-62	AAO89278, oxalate decarboxylase [Flammulina sp. IJF 140502], 68%, 4e-55 	B	N	
Oxalate decarboxylase type - 6	GU734365	EAA35545, oxalate decarboxylase oxdC [Neurospora crassa OR74A], 67%, 1e-55		A	A	
Oxalate decarboxylase type - 7	GU734366	EDQ99885, predicted protein [Laccaria bicolor S238N-H82], 67%, 1e-54	CAG34243, oxalate oxidase [Ceriporiopsis subvermispora], 52%, 1e-42 	B	A	
Oxalate decarboxylase type - 8	GU734367	EED81598, candidate oxalate decarboxylase [Postia placenta Mad-698-R], 61%, 2e-50		B	A	
Oxalate decarboxylase type - 9	GU734368	EAU85209, hypothetical protein CC1G_06225 [Coprinopsis cinerea okayama7#130], 69%, 2e-55	AAO89278, oxalate decarboxylase [Flammulina sp. IJF 140502], 64%, 7e-53 	B	A	
Oxalate decarboxylase type - 10	GU734369	EED81598, candidate oxalate decarboxylase [Postia placenta Mad-698-R], 65%, 4e-52		B	N	
Oxalate decarboxylase type - 11.1	GU734370	EAU85209, hypothetical protein CC1G_06225 [Coprinopsis cinerea okayama7#130], 74%, 8e-62	AAO89278, oxalate decarboxylase [Flammulina sp. IJF 140502], 66%, 2e-53 	B	N	
Oxalate decarboxylase type - 11.2	GU734371	EAU85209, hypothetical protein CC1G_06225 [Coprinopsis cinerea okayama7#130], 74%, 3e-61	AAO89278, oxalate decarboxylase [Flammulina sp. IJF 140502], 65%, 9e-53 	B	N	
Oxalate decarboxylase type - 12	GU734372	EAU85209, hypothetical protein CC1G_06225 [Coprinopsis cinerea okayama7#130], 76%, 1e-61	AAO89278, oxalate decarboxylase [Flammulina sp. IJF 140502], 73%, 6e-58 	B	N	
Oxalate decarboxylase type - 13	GU734373	EAU85209, hypothetical protein CC1G_06225 [Coprinopsis cinerea okayama7#130], 75%, 4e-62	AAO89278, oxalate decarboxylase [Flammulina sp. IJF 140502], 66%, 2e-54 	B	N	
Oxalate decarboxylase - type 14	GU734374	EAU85209, hypothetical protein CC1G_06225 [Coprinopsis cinerea okayama7#130], 64%, 2e-50	AAQ67425, oxalate decarboxylase [Trametes versicolor], 55%, 9e-43	B	N	
Tyrosinase - type 1	GU734550	BAF74396, tyrosinase [Pholiota nameko], 30%, 8e-10		B	A	
Tyrosinase - type 2	GU734551	BAE48755, polyphenoloxidase [Agaricus blazei], 52%, 4e-39		B	A	
Tyrosinase - type 3	GU734552	BAF74396, tyrosinase [Pholiota nameko], 40%, 6e-19		B	A	
Tyrosinase - type 4	GU734553	EDN17470, hypothetical protein BC1G_00048 [Botryotinia fuckeliana B05.10], 39%, 1e-21	EEQ33343, tyrosinase [Microsporum canis CBS 113480], 32%, 2e-17	A	N	
Tyrosinase - type 5	GU734554	EDN21791, hypothetical protein BC1G_14990 [Botryotinia fuckeliana B05.10], 52%, 6e-43	EEQ33343, tyrosinase [Microsporum canis CBS 113480], 34%, 3e-20	A	N	
Glycoside hydrolase family 3 – type 1	GU734375	EDN30805 hypothetical protein BC1G_10221 [Botryotinia fuckeliana B05.10], 85%, 1e-37	AAZ95588, thermostable beta-glucosidase [Thermoascus aurantiacus], 87%, 4e-37 	A	A	
Glycoside hydrolase family 3 – type 2	GU734376	EAA35949 hypothetical protein NCU08054 [Neurospora crassa OR74A], 74%, 1e-28	EAW08763, glycosyl hydrolase family 3 N terminal domain protein [Aspergillus clavatus NRRL 1], 75%, 3e-28	A	A	
Glycoside hydrolase family 3 – type 3	GU734377	EDN26263, hypothetical protein BC1G_06666 [Botryotinia fuckeliana B05.10], 83%, 4e-29	AAT95380, beta-glucosidase [Phaeosphaeria sp. S-93-48], 71%, 4e-27	A	A	
Glycoside hydrolase family 3 – type 4	GU734378	EAT85191, hypothetical protein SNOG_07725 [Phaeosphaeria nodorum SN15], 85%, 2e-34	EDU48263, beta-glucosidase 1 precursor [Pyrenophora tritici-repentis Pt-1C-BFP], 81%, 1e-32	A	A	
Glycoside hydrolase family 3 – type 5	GU734379	AAT95379, beta-glucosidase [Phaeosphaeria avenaria f. sp. triticae], 73%, 1e-29		A	A	
Glycoside hydrolase family 3 – type 6	GU734380	AAM94393, avenacinase [Talaromyces emersonii], 76%, 2e-32		A	A	
Glycoside hydrolase family 3 – type 7	GU734381	CAP73666, unnamed protein product [Podospora anserina], 64%, 2e-20	EDP49439, beta glucosidase, putative [Aspergillus fumigatus A1163], 60%, 2e-20	A	A	
Glycoside hydrolase family 3 – type 8	GU734382	CAP73666, unnamed protein product [Podospora anserina], 70%, 1e-26	EED54167, beta-glucosidase, putative [Aspergillus flavus NRRL3357], 67%, 9e-24	A	N	
Glycoside hydrolase family 3 – type 9.1	GU734383	EDN98397, hypothetical protein SS1G_13255 [Sclerotinia sclerotiorum 1980], 77%, 1e-28	AAZ95588, thermostable beta-glucosidase [Thermoascus aurantiacus], 73%, 3e-28 	A	A	
Glycoside hydrolase family 3 – type 9.2	GU734384	EDO04663 beta-glucosidase 1 precursor [Sclerotinia sclerotiorum 1980], 69%, 3e-29		A	A	
Glycoside hydrolase family 3 – type 10	GU734385	EAA61767, hypothetical protein AN7396.2 [Aspergillus nidulans FGSC A4], 67%, 2e-25	EAL84958, beta-glucosidase, putative [Aspergillus fumigatus Af293], 61%, 7e-22	A	A	
Glycoside hydrolase family 3 – type 11	GU734386	EDN30513, hypothetical protein BC1G_09832 [Botryotinia fuckeliana B05.10], 87%, 7e-36	EDU48263, beta-glucosidase 1 precursor [Pyrenophora tritici-repentis Pt-1C-BFP], 79%, 1e-31	A	A	
Glycoside hydrolase family 3 – type 12	GU734387	EDN30513, hypothetical protein BC1G_09832 [Botryotinia fuckeliana B05.10], 89%, 1e-36	EDU48263, beta-glucosidase 1 precursor [Pyrenophora tritici-repentis Pt-1C-BFP], 79%, 2e-32	A	A	
Glycoside hydrolase family 3 – type 13	GU734388	EAA57725, hypothetical protein AN5976.2 [Aspergillus nidulans FGSC A4], 69%, 3e-29	EED48455, beta-glucosidase 2 precursor, putative [Aspergillus flavus NRRL3357], 68%, 4e-28	A	A	
Glycoside hydrolase family 3 – type 14	GU734389	EAA35949 hypothetical protein NCU08054 [Neurospora crassa OR74A], 79%, 3e-32	EDU48263, beta-glucosidase 1 precursor [Pyrenophora tritici-repentis Pt-1C-BFP], 72%, 3e-29	A	A	
Glycoside hydrolase family 3 – type 15	GU734390	CAP73666, unnamed protein product [Podospora anserina], 72%, 5e-27	AAA91297, beta-glucosidase [Kuraishia capsulata], 72%, 7e-25	A	A,N	
Glycoside hydrolase family 3 – type 16	GU734391	EAA35949 hypothetical protein NCU08054 [Neurospora crassa OR74A], 84%, 4e-34	EDU48263, beta-glucosidase 1 precursor [Pyrenophora tritici-repentis Pt-1C-BFP], 79%, 3e-31	A	A	
Glycoside hydrolase family 3 – type 17	GU734392	EDN98397, hypothetical protein SS1G_13255 [Sclerotinia sclerotiorum 1980], 70%, 5e-27	EDU39662, beta-glucosidase 2 precursor [Pyrenophora tritici-repentis Pt-1C-BFP], 60%, 1e-23	A	A	
Glycoside hydrolase family 3 – type 18	GU734393	EDN98397, hypothetical protein SS1G_13255 [Sclerotinia sclerotiorum 1980], 82%, 4e-32	EDU39662, beta-glucosidase 2 precursor [Pyrenophora tritici-repentis Pt-1C-BFP], 77%, 3e-31	A	A	
Glycoside hydrolase family 3 – type 19	GU734394	EDN98397, hypothetical protein SS1G_13255 [Sclerotinia sclerotiorum 1980], 80%, 2e-29	EDO04663, beta-glucosidase 1 precursor [Sclerotinia sclerotiorum 1980], 75%, 2e-29	A	A	
Glycoside hydrolase family 3 – type 20	GU734395	ABS71124, beta-glucosidase [Penicillium occitanis], 69%, 1e-27		A	N	
Glycoside hydrolase family 3 – type 21	GU734396	ABP88968, beta-glucosidase [Penicillium brasilianum], 72%, 1e-27		A	A	
Glycoside hydrolase family 3 – type 22	GU734397	CAP61089, unnamed protein product [Podospora anserina], 82%, 4e-28	EEA22879, beta-D-glucoside glucohydrolase [Penicillium marneffei ATCC 18224], 78%, 6e-27	A	A	
Glycoside hydrolase family 3 – type 23	GU734398	CAP61089, unnamed protein product [Podospora anserina], 79%, 4e-27	EEA22879, beta-D-glucoside glucohydrolase [Penicillium marneffei ATCC 18224], 75%, 7e-26	A	A	
Glycoside hydrolase family 3 – type 24	GU734399	EAA59363, hypothetical protein AN4102.2 [Aspergillus nidulans FGSC A4], 73%, 1e-28	EED47060, beta-glucosidase, putative [Aspergillus flavus NRRL3357], 71%, 2e-28	A	N	
Glycoside hydrolase family 5 – type 1	GU734400	ABG79371, Man5C (mannanase) [Phanerochaete chrysosporium], 64%, 9e-36 		B	A	
Glycoside hydrolase family 5 – type 2	GU734401	EAQ87588, hypothetical protein CHGG_04207 [Chaetomium globosum CBS 148.51], 70%, 1e-41	1QNO_A, Chain A, The 3-D Structure Of A Trichoderma reesei B-Mannanase From Glycoside Hydrolase Family 5, 70%, 6e-41	A	A	
Glycoside hydrolase family 5 – type 3	GU734402	ABG79370, Man5D [Phanerochaete chrysosporium], 80%, 5e-48 		B	A	
Glycoside hydrolase family 5 – type 4	GU734403	ABG79370, Man5D [Phanerochaete chrysosporium], 71%, 4e-42 		B	A	
Glycoside hydrolase family 5 – type 5	GU734404	ABG79370, Man5D [Phanerochaete chrysosporium], 79%, 7e-48 		B	N	
Glycoside hydrolase family 5 – type 6	GU734405	EDN91343, hypothetical protein SS1G_00746 [Sclerotinia sclerotiorum 1980], 77%, 2e-46	EEA18972, endo-1,4-beta-mannosidase [Penicillium marneffei ATCC 18224], 68%, 7e-37	A	N	
Glycoside hydrolase family 5 – type 7	GU734406	ABG79370, Man5D [Phanerochaete chrysosporium], 77%, 8e-46 		B	N	
Glycoside hydrolase family 5 – type 8	GU734407	EDN91343, hypothetical protein SS1G_00746 [Sclerotinia sclerotiorum 1980], 91%, 2e-56	AAA67426, mannanase [Aspergillus aculeatus], 68%, 2e-36
	A	N	
Glycoside hydrolase family 5 – type 9	GU734408	CAB76904 CEL4a mannanase [Agaricus bisporus], 64% 1e-39 		B	N	
Glycoside hydrolase family 6 – type 1	GU734409	EED17133, cellobiohydrolase, putative [Talaromyces stipitatus ATCC 10500], 82%, 5e-61		A	A	
Glycoside hydrolase family 6 – type 2	GU734410	EDJ96050, hypothetical protein MGG_04499 [Magnaporthe grisea 70-15], 70%, 5e-53	EAA27345, exoglucanase 3 precursor [Neurospora crassa OR74A], 68%, 3e-51	A	A	
Glycoside hydrolase family 6 – type 3	GU734411	AAT64008, cellobiohydrolase II-I [Volvariella volvacea], 83%, 2e-62 		B	N	
Glycoside hydrolase family 7 – type 1.1	GU734555	AAF05699, exoglucanase [Alternaria alternata], 85%, 5e-83		A	A	
Glycoside hydrolase family 7 – type 1.2	GU734556	AAF05699, exoglucanase [Alternaria alternata], 86%, 1e-83		A	N	
Glycoside hydrolase family 7 – type 1.3	GU734557	AAF05699, exoglucanase [Alternaria alternata], 85%, 1e-82		A	A	
Glycoside hydrolase family 7 – type 1.4	GU734558	AAF05699, exoglucanase [Alternaria alternata], 86%, 5e-84		A	N	
Glycoside hydrolase family 7 – type 2	GU734559	EDN21446, hypothetical protein BC1G_14702 [Botryotinia fuckeliana B05.10], 76%, 5e-71	ABY61333, GH 7 family exocellobiohydrolase [Infundibulicybe gibba], 73%, 7e-70	A	A	
Glycoside hydrolase family 7 – type 3	GU734560	EDN21446, hypothetical protein BC1G_14702 [Botryotinia fuckeliana B05.10], 79%, 2e-74	AAL89553, cellobiohydrolase I catalytic domain [Talaromyces emersonii], 76%, 3e-72	A	A	
Glycoside hydrolase family 7 – type 4	GU734561	ABY61336, GH 7 family exocellobiohydrolase [Infundibulicybe gibba], 84%, 8e-78		B	N	
Glycoside hydrolase family 7 – type 5	GU734562	ABY61336, GH 7 family exocellobiohydrolase [Infundibulicybe gibba], 80%, 3e-75		B	N	
Glycoside hydrolase family 7 – type 6	GU734563	BAC07255, cellobiohydrolase C [Aspergillus oryzae], 74%, 9e-72		A	A	
Glycoside hydrolase family 7 – type 7	GU734564	CAA80253, cellulase [Phanerochaete chrysosporium], 79%, 2e-75		B	N	
Glycoside hydrolase family 7 – type 8	GU734565	AAF05699, exoglucanase [Alternaria alternata], 86%, 5e-84		A	N	
Glycoside hydrolase family 10 – type 1	GU734412	EDN91106, hypothetical protein SS1G_00509 [Sclerotinia sclerotiorum 1980], 76%, 4e-53	BAB69073, xylanseF3 [Aspergillus oryzae], 73%, 6e-53 	A	A	
Glycoside hydrolase family 10 – type 2	GU734413	EDN91106, hypothetical protein SS1G_00509 [Sclerotinia sclerotiorum 1980], 78%, 2e-56	BAA92882, endo-1,4 beta-D-xylanase [Aspergillus sojae], 72%, 3e-53 	A	A	
Glycoside hydrolase family 10 – type 3	GU734414	CAC03463, putative xylanase [Agaricus bisporus], 76%, 9e-57		B	N	
Glycoside hydrolase family 11 – type 1.1	GU734415	EDO01144, hypothetical protein SS1G_03618 [Sclerotinia sclerotiorum 1980], 77%, 1e-54	ACF40831, endo-1,4-beta-xylanase [Hypocrea lixii], 75%, 5e-52	A	A,N	
Glycoside hydrolase family 11 – type 1.2	GU734416	EDO01144, hypothetical protein SS1G_03618 [Sclerotinia sclerotiorum 1980], 77%, 1e-54	ACF40831, endo-1,4-beta-xylanase [Hypocrea lixii], 75%, 5e-52	A	A,N	
Glycoside hydrolase family 11 – type 1.3	GU734417	EDO01144, hypothetical protein SS1G_03618 [Sclerotinia sclerotiorum 1980], 77%, 1e-54	ACF40831, endo-1,4-beta-xylanase [Hypocrea lixii], 75%, 5e-52	A	A	
Glycoside hydrolase family 11 – type 2	GU734418	EDO01144, hypothetical protein SS1G_03618 [Sclerotinia sclerotiorum 1980], 76%, 5e-55	ACP27609.1|  endo-1,4-beta-xylanase [Penicillium canescens], 74%, 2e-52	A	A	
Glycoside hydrolase family 11 – type 3	GU734419	AAG44995, endo-1,4-B-xylanase B [Phanerochaete chrysosporium], 76%, 1e-52		B	A	
Glycoside hydrolase family 11 – type 4.1	GU734420	AAB29056, xylanase A {EC 3.2.1.8} [Schizophyllum commune], 83%, 7e-64 		B	N	
Glycoside hydrolase family 11 – type 4.2	GU734421	AAB29056, xylanase A {EC 3.2.1.8} [Schizophyllum commune], 83%, 1e-63		B	N	
Glycoside hydrolase family 11 – type 5	GU734422	ABN81018, xylanase [Leucoagaricus gongylophorus], 83%, 8e-60 		B	A,N	
Glycoside hydrolase family 11 – type 6	GU734423	EDN20200, hypothetical protein BC1G_03590 [Botryotinia fuckeliana B05.10], 77%, 4e-55	ABG37634, XYN5 [Acrophialophora nainiana], 76%, 3e-53 	A	N	
Glycoside hydrolase family 11 – type 7	GU734424	EDO01144, hypothetical protein SS1G_03618 [Sclerotinia sclerotiorum 1980], 79%, 1e-56	BAE71133, endo-1,4-beta-xylanase [Penicillium citrinum], 75%, 5e-54 	A	N	
Glycoside hydrolase family 11 – type 8.1	GU734425	EDO01144, hypothetical protein SS1G_03618 [Sclerotinia sclerotiorum 1980], 82%, 2e-57	CAA93120, endo-1,4-betaxylanase [Ascochyta pisi], 75%, 1e-53	A	A	
Glycoside hydrolase family 11 – type 8.2	GU734426	EDO01144, hypothetical protein SS1G_03618 [Sclerotinia sclerotiorum 1980], 82%, 4e-57	CAA93120, endo-1,4-betaxylanase [Ascochyta pisi], 74%, 4e-53	A	A	
Glycoside hydrolase family 11 – type 9	GU734427	AAB06573, endo-beta-1,4-D-xylanase [Magnaporthe grisea], 69%, 9e-51		A	A,N	
Glycoside hydrolase family 11 – type 10	GU734428	EED48943, endo-1,4-beta-xylanase (XlnA), putative [Aspergillus flavus NRRL3357], 76%, 2e-50		A	A	
Glycoside hydrolase family 18 – type 1	GU734429	CAC35202, endochitinase [Amanita muscaria], 74%, 6e-36		B	A	
Glycoside hydrolase family 18 – type 2.1	GU734430	EDR01922, glycoside hydrolase family 18 protein [Laccaria bicolor S238N-H82], 68%, 6e-32		B	N	
Glycoside hydrolase family 18 – type 2.2	GU734431	EDR01921, glycoside hydrolase family 18 protein [Laccaria bicolor S238N-H82], 67%, 4e-31		B	N	
Glycoside hydrolase family 18 – type 3	GU734432	CAC35202, endochitinase [Amanita muscaria], 65%, 3e-32		B	N	
Glycoside hydrolase family 18 – type 4	GU734433	EED79911, hypothetical protein POSPLDRAFT_118230 [Postia placenta Mad-698-R], 53%, 9e-21	CAC35202, endochitinase [Amanita muscaria], 47%, 2e-20	B	A,N	
Glycoside hydrolase family 18 – type 5	GU734434	XP_383388, hypothetical protein FG03212.1 [Gibberella zeae PH-1], 66%, 6e-31	CAQ51152, chitinase [Coprinellus congregatus], 61%, 3e-30	A	N	
Glycoside hydrolase family 18 – type 6	GU734435	CAC35202, endochitinase [Amanita muscaria], 68%, 3e-32		B	N	
Glycoside hydrolase family 18 – type 7	GU734436	AAF47562, chitinase 2 [Drosophila melanogaster], 65%, 4e-29		-	A	
Glycoside hydrolase family 18 – type 8	GU734437	EDR01922, glycoside hydrolase family 18 protein [Laccaria bicolor S238N-H82], 72%, 1e-32		B	N	
Glycoside hydrolase family 18 – type 9	GU734438	EEB93366, hypothetical protein MPER_07986 [Moniliophthora perniciosa FA553], 64%, 4e-29	CAC35202, endochitinase [Amanita muscaria], 63%, 2e-28	B	A	
Glycoside hydrolase family 18 – type 10	GU734439	EAU84319, hypothetical protein CC1G_01315 [Coprinopsis cinerea okayama7#130], 69%, 6e-29	CAQ51152, chitinase [Coprinellus congregatus], 64%, 1e-28	B	N	
Glycoside hydrolase family 18 – type 11	GU734440	EAU84319, hypothetical protein CC1G_01315 [Coprinopsis cinerea okayama7#130], 67%, 9e-32	CAQ51152, chitinase [Coprinellus congregatus], 65%, 4e-31	B	N	
Glycoside hydrolase family 20 – type 1.1	GU734441	EEB94073, hypothetical protein MPER_07179 [Moniliophthora perniciosa FA553], 82%, 1e-44	EED78592, N-acetylhexosaminidase [Postia placenta Mad-698-R], 51%, 7e-26	B	N	
Glycoside hydrolase family 20 – type 1.2	GU734442	EEB94073, hypothetical protein MPER_07179 [Moniliophthora perniciosa FA553], 83%, 5e-45	EED78592, N-acetylhexosaminidase [Postia placenta Mad-698-R], 52%, 3e-26	B	N	
Glycoside hydrolase family 20 – type 2	GU734443	ABN66755, Mannosyl-glycoprotein endo-beta-N-acetylglucosamidase [Pichia stipitis CBS 6054], 36%, 4e-11		A	A	
Glycoside hydrolase family 20 – type 3	GU734444	EEB94073, hypothetical protein MPER_07179 [Moniliophthora perniciosa FA553], 61%, 3e-31	EED84038, N-acetylhexosaminidase [Postia placenta Mad-698-R], 58%, 9e-31	B	A	
Glycoside hydrolase family 20 – type 4	GU734445	EED84038, N-acetylhexosaminidase [Postia placenta Mad-698-R], 59%, 2e-31		B	N	
Glycoside hydrolase family 20 – type 5	GU734446	EEB94073, hypothetical protein MPER_07179 [Moniliophthora perniciosa FA553], 63%, 2e-32	EED84038, N-acetylhexosaminidase [Postia placenta Mad-698-R], 61%, 2e-31	B	A	
Glycoside hydrolase family 20 – type 6	GU734447	EDR13643, glycoside hydrolase family 20 protein [Laccaria bicolor S238N-H82], 65%, 2e-35		B	A	
Glycoside hydrolase family 20 – type 7	GU734448	EDR13643, glycoside hydrolase family 20 protein [Laccaria bicolor S238N-H82], 73%, 7e-38		B	A	
Glycoside hydrolase family 20 – type 8	GU734449	EDR13643, glycoside hydrolase family 20 protein [Laccaria bicolor S238N-H82], 71%, 9e-34		B	A,N	
Glycoside hydrolase family 20 – type 9	GU734450	EEH16325, beta-hexosaminidase [Paracoccidioides brasiliensis Pb03], 32%, 4e-09		A	A	
Glycoside hydrolase family 20 – type 10	GU734451	EDR13643, glycoside hydrolase family 20 protein [Laccaria bicolor S238N-H82], 72%, 5e-37		B	A,N	
Glycoside hydrolase family 20 – type 11	GU734452	EDR13643, glycoside hydrolase family 20 protein [Laccaria bicolor S238N-H82], 72%, 2e-36		B	N	
Glycoside hydrolase family 20 – type 12	GU734453	EED82341, N-acetylhexosaminidase [Postia placenta Mad-698-R], 39%, 1e-17		B	N	
Glycoside hydrolase family 20 – type 13	GU734454	EDU44531, beta-hexosaminidase beta chain precursor [Pyrenophora tritici-repentis 
Pt-1C-BFP], 58%, 9e-29		A	A	
Glycoside hydrolase family 20 – type 14	GU734455	EDR13643, glycoside hydrolase family 20 protein [Laccaria bicolor S238N-H82], 64%, 5e-31		B	N	
Glycoside hydrolase family 30 – type 1	GU734456	EAW15357, beta-1,6-glucanase, putative [Aspergillus clavatus NRRL 1], 76%, 2e-69		A	A	
Glycoside hydrolase family 30 – type 2	GU734457	BAB91213, beta-1,6-glucanase Neg1 [Neurospora crassa], 71%, 1e-59 		A	N	
Glycoside hydrolase family 31 – type 1	GU734458	EDU47577, alpha-glucosidase precursor [Pyrenophora tritici-repentis Pt-1C-BFP], 63%, 4e-121		A	A	
Glycoside hydrolase family 31 – type 2	GU734459	EDN19085, hypothetical protein BC1G_12859 [Botryotinia fuckeliana B05.10], 72%, 2e-137	EAA36478, alpha-glucosidase precursor [Neurospora crassa OR74A], 64%, 6e-123	A	A	
Glycoside hydrolase family 31 – type 3	GU734460	EDN19085, hypothetical protein BC1G_12859 [Botryotinia fuckeliana B05.10], 72%, 9e-136	EAA36478, alpha-glucosidase precursor [Neurospora crassa OR74A], 65%, 4e-124	A	A	
Glycoside hydrolase family 31 – type 4	GU734461	EDU47577, alpha-glucosidase precursor [Pyrenophora tritici-repentis Pt-1C-BFP], 69%, 6e-132		A	A	
Glycoside hydrolase family 31 – type 5	GU734462	EAW10465, alpha-glucosidase AgdA, putative [Aspergillus clavatus NRRL 1], 67%, 6e-128		A	N	
Glycoside hydrolase family 31 – type 6	GU734463	EEH06911, alpha-glucosidase [Ajellomyces capsulatus G186AR], 57%, 9e-111		A	N	
Glycoside hydrolase family 31 – type 7	GU734464	EDN19085, hypothetical protein BC1G_12859 [Botryotinia fuckeliana B05.10], 68%, 7e-132	EAA36478, alpha-glucosidase precursor [Neurospora crassa OR74A], 65%, 3e-127	A	N	
Glycoside hydrolase family 31 – type 8	GU734465	EAW25102, alpha-glucosidase AgdA, putative [Neosartorya fischeri NRRL 181], 66%, 1e-124		A	N	
Glycoside hydrolase family 31 – type 9	GU734466	EDU47577, alpha-glucosidase precursor [Pyrenophora tritici-repentis Pt-1C-BFP], 69%, 2e-133		A	N	
Glycoside hydrolase family 31 – type 10	GU734467	EDU47577, alpha-glucosidase precursor [Pyrenophora tritici-repentis Pt-1C-BFP], 67%, 6e-133		A	N	
Glycoside hydrolase family 45 – type 1.1	GU734468	EAT81011, hypothetical protein SNOG_11303 [Phaeosphaeria nodorum SN15], 83%, 4e-57	EDU47736, endoglucanase-5 [Pyrenophora tritici-repentis Pt-1C-BFP], 77%, 1e-53	A	A,N	
Glycoside hydrolase family 45 – type 1.2	GU734469	EAT81011, hypothetical protein SNOG_11303 [Phaeosphaeria nodorum SN15], 83%, 4e-57	EDU47736, endoglucanase-5 [Pyrenophora tritici-repentis Pt-1C-BFP], 77%, 1e-53	A	A	
Glycoside hydrolase family 45 – type 2	GU734470	EAT81011, hypothetical protein SNOG_11303 [Phaeosphaeria nodorum SN15], 85%, 1e-57	EDU47736, endoglucanase-5 [Pyrenophora tritici-repentis Pt-1C-BFP], 77%, 7e-54	A	A,N	
Glycoside hydrolase family 45 – type 3	GU734471	EAT81011, hypothetical protein SNOG_11303 [Phaeosphaeria nodorum SN15], 86%, 3e-58	EDU47736, endoglucanase-5 [Pyrenophora tritici-repentis Pt-1C-BFP], 78%, 2e-54	A	A,N	
Glycoside hydrolase family 45 – type 4	GU734472	ABU49185, endoglucanase [Syncephalastrum racemosum], 62%, 9e-39 		Z	N	
Glycoside hydrolase family 45 – type 5	GU734473	ABU49185, endoglucanase [Syncephalastrum racemosum], 63%, 1e-40 		Z	N	
Glycoside hydrolase family 45 – type 6	GU734474	EDN96900, hypothetical protein SS1G_01828 [Sclerotinia sclerotiorum 1980], 80%, 4e-52	AAR02399, glycoside hydrolase 45 [Gibberella zeae], 64%, 1e-41	A	A	
Glycoside hydrolase family 45 – type 7	GU734475	AAR02399, glycoside hydrolase 45 [Gibberella zeae], 63%, 2e-38		A	N	
Glycoside hydrolase family 45 – type 8	GU734476	BAD95808, endo-beta-D-1,4-glucanase [Mucor circinelloides], 66%, 5e-41 		Z	A	
Glycoside hydrolase family 45 – type 9	GU734477	EDN99000, hypothetical protein SS1G_13860 [Sclerotinia sclerotiorum 1980], 73%, 4e-46	EAA27871, endoglucanase V [Neurospora crassa OR74A], 65%, 5e-42	A	N	
Glycoside hydrolase family 45 – type 10	GU734478	EDN96900, hypothetical protein SS1G_01828 [Sclerotinia sclerotiorum 1980], 69%, 7e-46	AAR02399, glycoside hydrolase 45 [Gibberella zeae], 64%, 8e-40	A	A	
Glycoside hydrolase family 51 – type 1.1	GU734479	EED85858, predicted protein [Postia placenta Mad-698-R], 71%, 2e-23	EAL93812, alpha-L-arabinofuranosidase A [Aspergillus fumigatus Af293], 54%, 1e-18	B	A	
Glycoside hydrolase family 51 – type 1.2	GU734480	EED85858, predicted protein [Postia placenta Mad-698-R], 71%, 2e-23	EAL93812, alpha-L-arabinofuranosidase A [Aspergillus fumigatus Af293], 54%, 1e-18	B	N	
Glycoside hydrolase family 51 – type 2	GU734481	EED85858, predicted protein [Postia placenta Mad-698-R], 68%, 1e-23	ABO93602, alpha-L-arabinofuranosidase 2 [Penicillium purpurogenum], 49%, 4e-17 	B	A	
Glycoside hydrolase family 51 – type 3	GU734482	EED85858, predicted protein [Postia placenta Mad-698-R], 78%, 8e-30	ABO93602, alpha-L-arabinofuranosidase 2 [Penicillium purpurogenum], 52%, 7e-18 	B	A	
Glycoside hydrolase family 51 – type 4.1	GU734483	EED85858, predicted protein [Postia placenta Mad-698-R], 76%, 1e-28	EAL93812, alpha-L-arabinofuranosidase A [Aspergillus fumigatus Af293], 53%, 1e-20	B	A	
Glycoside hydrolase family 51 – type 4.2	GU734484	EED85858, predicted protein [Postia placenta Mad-698-R], 74%, 3e-28	ABO93602, alpha-L-arabinofuranosidase 2 [Penicillium purpurogenum], 54%, 1e-20 	B	A	
Glycoside hydrolase family 51 – type 4.3	GU734485	EED85858, predicted protein [Postia placenta Mad-698-R], 74%, 3e-28	ABO93602, alpha-L-arabinofuranosidase 2 [Penicillium purpurogenum], 54%, 1e-20 	B	N	
Glycoside hydrolase family 51 – type 4.4	GU734486	EED85858, predicted protein [Postia placenta Mad-698-R], 78%, 5e-29	ABO93602, alpha-L-arabinofuranosidase 2 [Penicillium purpurogenum], 56%, 2e-21 	B	N	
Glycoside hydrolase family 51 – type 5	GU734487	EED85858, predicted protein [Postia placenta Mad-698-R], 74%, 6e-28	ABO93602, alpha-L-arabinofuranosidase 2 [Penicillium purpurogenum], 50%, 1e-17 	B	N	
Glycoside hydrolase family 67 – type 1	GU734488	EEA23405, alpha-glucuronidase precursor, putative [Penicillium marneffei ATCC 18224, 75%, 6e-82		A	A	
Glycoside hydrolase family 67 – type 2	GU734489	EAW17337, alpha-glucuronidase [Neosartorya fischeri NRRL 181], 78%, 1e-84		A	A	
Glycoside hydrolase family 67 – type 3	GU734490	EDU45681, alpha glucuronidase [Pyrenophora tritici-repentis Pt-1C-BFP], 77%, 9e-86		A	A	
Glycoside hydrolase family 67 – type 4	GU734491	EEA20307, alpha-glucuronidase [Penicillium marneffei ATCC 18224], 79%, 8e-85		A	A	
Glycoside hydrolase family 67 – type 5	GU734492	EAW13280, alpha-glucuronidase [Aspergillus clavatus NRRL 1], 85%, 4e-90		A	A	
Glycoside hydrolase family 67 – type 6	GU734493	EEA20307, alpha-glucuronidase [Penicillium marneffei ATCC 18224], 75%, 1e-81		A	N	
Glycoside hydrolase family 67 – type 7	GU734494	EEA20307, alpha-glucuronidase [Penicillium marneffei ATCC 18224], 77%, 6e-83		A	N	
Glycoside hydrolase family 67 – type 8	GU734495	EEA20307, alpha-glucuronidase [Penicillium marneffei ATCC 18224], 76%, 4e-83		A	N	
Glycoside hydrolase family 74 – type 1	GU734496	EEB90475, hypothetical protein MPER_11313 [Moniliophthora perniciosa FA553], 72%, 4e-61	BAF95189, glycoside hydrolase family 74 [Phanerochaete chrysosporium], 71%, 2e-59	B	A	
Glycoside hydrolase family 74 – type 2	GU734497	CAC02964, CEL6 protein [Agaricus bisporus], 71%, 6e-60	EAL85019, endoglucanase, putative [Aspergillus fumigatus Af293], 72%, 1e-55	B	A	
Glycoside hydrolase family 74 – type 3	GU734498	CAC02964, CEL6 protein [Agaricus bisporus], 65%, 1e-55		B	N	
Glycoside hydrolase family 74 – type 4	GU734499	CAC02964, CEL6 protein [Agaricus bisporus], 65%, 7e-55		B	N	
Glycoside hydrolase family 92 – type 1	GU734500	EDN97094, hypothetical protein SS1G_02022 [Sclerotinia sclerotiorum 1980], 83%, 5e-32	EED23103, alpha-1,2-mannosidase family protein, putative [Talaromyces stipitatus ATCC 10500], 79%, 1e-31	A	A,N	
Glycoside hydrolase family 92 – type 2	GU734501	EAW07756, alpha-1,2-mannosidase family protein, putative [Aspergillus clavatus NRRL 1], 82%, 3e-32		A	A	
Glycoside hydrolase family 92 – type 3	GU734502	EEH16737, alpha-1,2-mannosidase family protein [Paracoccidioides brasiliensis Pb03], 87%, 1e-33		A	N	
Glycoside hydrolase family 92 – type 4	GU734503	EEH16737, alpha-1,2-mannosidase family protein [Paracoccidioides brasiliensis Pb03], 89%, 9e-34		A	N	
Glycoside hydrolase family 92 – type 5	GU734504	EDN25459, hypothetical protein BC1G_01019 [Botryotinia fuckeliana B05.10], 87%, 1e-34	EAW16623, alpha-1,2-mannosidase, putative subfamily [Neosartorya fischeri NRRL 181], 82%, 2e-33	A	N	
Glycoside hydrolase family 92 – type 6	GU734505	EEH16737, alpha-1,2-mannosidase family protein [Paracoccidioides brasiliensis Pb03], 89%, 4e-34		A	N	
Glycoside hydrolase family 92 – type 7	GU734506	EEH16737, alpha-1,2-mannosidase family protein [Paracoccidioides brasiliensis Pb03], 73%, 6e-27		A	N	
Glycoside hydrolase family 92 – type 8	GU734507	EAA31188, hypothetical protein NCU04798 [Neurospora crassa OR74A], 82%, 1e-32	EEA25030, alpha-1,2-mannosidase, putative [Penicillium marneffei ATCC 18224], 71%, 3e-30	A	N	
Glycoside hydrolase family 92 – type 9	GU734508	EEH16737, alpha-1,2-mannosidase family protein [Paracoccidioides brasiliensis Pb03], 86%, 7e-33		A	N	
Glycoside hydrolase family 114 – type 1	GU734509	XP_390061, hypothetical protein FG09885.1 [Gibberella zeae PH-1], 67%, 3e-55	EDU47645, endo alpha-1,4 polygalactosaminidase precusor [Pyrenophora tritici-repentis Pt-1C-BFP], 62%, 1e-52	A	A	
Glycoside hydrolase family 114 – type 2	GU734510	XP_390061, hypothetical protein FG09885.1 [Gibberella zeae PH-1], 57%, 2e-45	EDU47645, endo alpha-1,4 polygalactosaminidase precusor [Pyrenophora tritici-repentis Pt-1C-BFP], 51%, 2e-43	A	A	
Glycoside hydrolase family 114 – type 3	GU734511	XP_390061, hypothetical protein FG09885.1 [Gibberella zeae PH-1], 62%, 4e-51	EDU47645, endo alpha-1,4 polygalactosaminidase precusor [Pyrenophora tritici-repentis Pt-1C-BFP], 58%, 1e-49	A	A	
Glycoside hydrolase family 114 – type 4	GU734512	XP_390061, hypothetical protein FG09885.1 [Gibberella zeae PH-1], 49%, 2e-38	EED13654, endo alpha-1,4 polygalactosaminidase, putative [Talaromyces stipitatus ATCC 10500], 48%, 6e-37	A	A	
Glycoside hydrolase family 114 – type 5	GU734513	XP_390061, hypothetical protein FG09885.1 [Gibberella zeae PH-1], 47%, 9e-37	EED13654, endo alpha-1,4 polygalactosaminidase, putative [Talaromyces stipitatus ATCC 10500], 46%, 	A	A	
Glycoside hydrolase family 114 – type 6	GU734514	EDU46110, endo alpha-1,4 polygalactosaminidase precusor [Pyrenophora tritici-repentis Pt-1C-BFP], 47%, 4e-36		A	A	
Glycoside hydrolase family 114 – type 7	GU734515	EAT78463, hypothetical protein SNOG_14226 [Phaeosphaeria nodorum SN15], 72%, 3e-58	EDU47645, endo alpha-1,4 polygalactosaminidase precusor [Pyrenophora tritici-repentis Pt-1C-BFP], 70%, 5e-58	A	N	
Glycoside hydrolase family 114 – type 8	GU734516	EAT88101, hypothetical protein SNOG_04341 [Phaeosphaeria nodorum SN15], 56%, 1e-41	EDU47645, endo alpha-1,4 polygalactosaminidase precusor [Pyrenophora tritici-repentis Pt-1C-BFP], 50%, 4e-41	A	N	
Glycoside hydrolase family 114 – type 9	GU734517	CAP80405, Pc12g07780 [Penicillium chrysogenum Wisconsin 54-1255], 63%, 2e-53	EED58020, endo alpha-1,4 polygalactosaminidase, putative [Aspergillus flavus NRRL3357], 58%, 1e-49	A	N	
Glycoside hydrolase family 114 – type 10	GU734518	XP_390061, hypothetical protein FG09885.1 [Gibberella zeae PH-1], 63%, 1e-52	EDU47645, endo alpha-1,4 polygalactosaminidase precusor [Pyrenophora tritici-repentis Pt-1C-BFP], 58%, 9e-49	A	N	
Carbohydrate esterase family 1 (FGH) – type 1	GU734519	EDU49917, S-formylglutathione hydrolase [Pyrenophora tritici-repentis Pt-1C-BFP], 85%, 4e-34		A	A	
Carbohydrate esterase family 1 (FGH) – type 2	GU734520	CAP69126, unnamed protein product [Podospora anserina], 62%, 2e-19	EDN08165, esterase D [Ajellomyces capsulatus NAm1], 60%, 5e-18	A	A	
Carbohydrate esterase family 1 (FGH) – type 3	GU734521	CAG82371, YALI0C20405p [Yarrowia lipolytica], 76%, 7e-28	EEA21401, esterase, putative [Penicillium marneffei ATCC 18224], 67%, 5e-26	A	A	
Carbohydrate esterase family 1 (FGH) – type 4	GU734522	EDU49917, S-formylglutathione hydrolase [Pyrenophora tritici-repentis Pt-1C-BFP], 87%, 2e-34		A	A,N	
Carbohydrate esterase family 1 (FGH) – type 5	GU734523	EAW12859, esterase, putative [Aspergillus clavatus NRRL 1], 85%, 2e-35		A	N	
Carbohydrate esterase family 1 (FGH) – type 6	GU734524	EDU49917, S-formylglutathione hydrolase [Pyrenophora tritici-repentis Pt-1C-BFP], 98%, 1e-39		A	N	
Carbohydrate esterase family 1 (FGH) – type 7	GU734525	EDU49917, S-formylglutathione hydrolase [Pyrenophora tritici-repentis Pt-1C-BFP], 84%, 1e-32		A	N	
Carbohydrate esterase family 1 (FGH) – type 8	GU734526	EDU49917, S-formylglutathione hydrolase [Pyrenophora tritici-repentis Pt-1C-BFP], 83%, 7e-32		A	N	
Carbohydrate esterase family 1 (AXE) – type 1	GU734536	EAT76950, hypothetical protein SNOG_15575 [Phaeosphaeria nodorum SN15], 72%, 9e-35	EED52855, acetyl xylan esterase, putative [Aspergillus flavus NRRL3357], 70%, 7e-34	A	A	
Carbohydrate esterase family 1 (AXE) – type 2	GU734537	EED23004, acetyl xylan esterase, putative [Talaromyces stipitatus ATCC 10500], 66%, 2e-30		A	A	
Carbohydrate esterase family 1 (AXE) – type 3	GU734538	EAA35308, hypothetical protein NCU00710 [Neurospora crassa OR74A], 70%, 2e-33	EED23004, acetyl xylan esterase, putative [Talaromyces stipitatus ATCC 10500], 67%, 9e-33	A	N	
Carbohydrate esterase family 1 (AXE) – type 4	GU734539	EDU48221, acetylxylan esterase 1 precursor [Pyrenophora tritici-repentis Pt-1C-BFP], 68%, 1e-29		A	N	
Carbohydrate esterase family 1 (AXE) – type 5	GU734540	EAT76950, hypothetical protein SNOG_15575 [Phaeosphaeria nodorum SN15], 66%, 2e-29	EDU48221, acetylxylan esterase 1 precursor [Pyrenophora tritici-repentis Pt-1C-BFP], 64%, 7e-28	A	N	
Carbohydrate esterase family 9 – type 1.1	GU734527	EDR08790, carbohydrate esterase family 9 protein [Laccaria bicolor S238N-H82], 88%, 6e-40	ABF43501, amidohydrolase [Acidobacteria bacterium Ellin345], 42%, 8e-09	B	A	
Carbohydrate esterase family 9 – type 1.2	GU734528	EDR08790, carbohydrate esterase family 9 protein [Laccaria bicolor S238N-H82], 87%, 1e-39		B	A,N	
Carbohydrate esterase family 9 – type 2.1	GU734529	EED79062, candidate carbohydrate esterase protein from family CE9 [Postia placenta Mad-698-R], 73%, 1e-34		B	N	
Carbohydrate esterase family 9 – type 2.2	GU734530	EED79062, candidate carbohydrate esterase protein from family CE9 [Postia placenta Mad-698-R], 69%, 2e-30		B	N	
Carbohydrate esterase family 9 – type 3	GU734531	EDQ99532, carbohydrate esterase family 9 protein [Laccaria bicolor S238N-H82], 86%, 5e-39		B	A	
Carbohydrate esterase family 9 – type 4	GU734532	EDQ99532, carbohydrate esterase family 9 protein [Laccaria bicolor S238N-H82], 86%, 1e-40		B	A	
Carbohydrate esterase family 9 – type 5	GU734533	EED79062, candidate carbohydrate esterase protein from family CE9 [Postia placenta Mad-698-R], 73%, 2e-32		B	A	
Carbohydrate esterase family 9 – type 6	GU734534	EAU84370, hypothetical protein CC1G_01366 [Coprinopsis cinerea okayama7#130], 73%, 1e-33	EED79062, candidate carbohydrate esterase protein from family CE9 [Postia placenta Mad-698-R], 68%, 3e-32	B	A	
Carbohydrate esterase family 9 – type 7	GU734535	EDQ99532, carbohydrate esterase family 9 protein [Laccaria bicolor S238N-H82], 91%, 3e-43		B	A	
